# The Study of the Inhibition of the Recombinant TACE Prodomain to Endotoxemia in Mice

**DOI:** 10.3390/ijms10125442

**Published:** 2009-12-18

**Authors:** Xiaoou Li, Yuan Yan, Wei Huang, Yuzhen Yang

**Affiliations:** 1 Department of Pediatrics, Renming Hospital, Wuhan University, Wuhan 430044, China; E-Mail: lxou27@hotmail.com; 2 Department of Biochemistry and Molecular Biology, Tongji Medical College, Huazhong University of Science & Technology, Wuhan 430030, China; E-Mails: Yanyuan@hotmail.com (Y.Y.); huangwei@hotmail.com (W.H.)

**Keywords:** TACE, TNF-α, prodomain, inhibitor

## Abstract

**Objective:**

To demonstrate the inhibitory function of the prodomain of tumor necrosis factor-α (TNF-α) converting enzyme (TACE) on TACE activity and to develop an approach to interfere with inflammation processes.

**Methods:**

The cDNA encoding the full-length ectodomain (T1300) and prodomain (T591) of TACE were amplified by RT-PCR. The expression plasmids (pET-28a (+)-T1300 and pET-28a (+)-T591) were constructed and transformed into *E. coli* BL21. After Ni^2+^-NTA resin affinity chromatography, the recombinant T591 protein was obtained and assayed. In order to detect its inhibiton of TACE activity, the mice in the LPS-induced endotoxemia model group were treated with the recombinant TACE prodomain protein prior to the injection of LPS. Murine peritoneal macrophages were isolated from mice abdominal cavity for FCM and the liver, kidney and lung were removed for traditionally histopathology sectioning.

**Results:**

The FCM results showed that the recombinant prodomain protein decreased the release of the sTNF-α, which mediated the accumulation of TNF-α on the surface of macrophage cells. HE staining proved that the recombinant protein can decrease the inflammatory response in internal organs of endotoxaemia mice.

**Conclusions:**

The recombinant prodomain of TACE has the ability to inhibit sTNF-α release, which indicates that prodomain is an effective antagonist of TACE and might be useful in the molecular design of anti-inflammatory drugs.

## 1. Introduction

**Figure 1.**
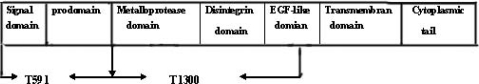
Structure of TACE.

The ADAMs (a disintergrin and metalloprotease) are a family of membrane-anchored glycoproteins that play an important role in development, cell-cell interaction, and protein ectodomain shedding [1]. ADAMs are usually comprised of several different proteins modules as follows: an *N*-terminal signal sequence, prodomain, a metalloprotease domain, a disintergrin domain, a cysteine-rich domain, an EGF-like domain, a transmembrane domain, and a cytoplasmic tail [2,3]. A conserved cysteine residue in the prodomain preferentially coordinates the essential active site zinc atom of the metalloprotease domain, keeping ADAMs in the latent state [4,5]. Proprotein convertases (PCs) cleave the prodomain from the rest of the protein at a conserved RxR motif, effectively releasing the prodomain and switching the zinc coordination to the metalloprotease domain, thereby making it available for catalytic activity [6,7]. The ADAMs metalloprotease contains a catalytic site consensus sequence (HE*XX*H). However, only a subset of the ADAMs, including ADAM17, contains this motif which is required for potentially active metalloproteases (Figure 1) [8,9].

The tumor necrosis factor-α converting enzyme (TACE, ADAM17) was purified on the basis of its ability to cleave tumor necrosis factor-α [10–12]. TACE is the key protease responsible for processing TNF-α from the 26 KD membrane anchored precursor (mTNF-α) to the secreted, 17 KD TNF-α (sTNF-α) [13,14]. T-lymphocytes from TACE knock-out mice produce 90% less secreted sTNF-α than wild type [10]. As is known, there is large difference between the biological activities of mTNF-α and sTNF-α, and the overproduction of sTNF-α plays a central role in the inflammatory process. sTNF-α is rapidly produced by macrophages in response to infectious challenges, and augments the phagocytic and cytotoxic actions of macrophages, as well as triggering the synthesis and secretion of proinflammatory mediators, including IL-1, GM-CSF, TNF itself, and other cytokines [15,16]. In a word, sTNF-α was involved in various pathophysiology inflammation processes. As mentioned above, TACE is considered to be an important sheddase for TNF-α, so an inhibitor of TACE has the ability to inhibit the cleavage process of mTNF-α and then suppress the production of sTNF-α, and might be used to prevent sTNF-α-releated diseases [17,18].

The prodomain of ADAM17 contains a cysteine switch sequence that presumably keeps ADAM17 in latent form similar to other ADAMs members [19]. On the basis of this hypothesis, we presumed that the prodomain has the ability to maintain TACE latency. In this study, we investigated the inhibitory function of the prodomain on TACE catalytic activity, using a well-established LPS-induced endotoxemia model. With this model, we hope to develop a new approach to interfere inflammation processes.

## 2. Results and Discussion

### 2.1. Expression and Purification of TACE Prokaryotic Expression Plasmids

The prokaryotic expression plasmids (pET-28a-T591 and pET-28a-T1300) were constructed successfully. T591 and T1300 DNA fragments contain 591bp and about 1300bp respectively, and they were also confirmed by sequencing.

**Figure 2.**
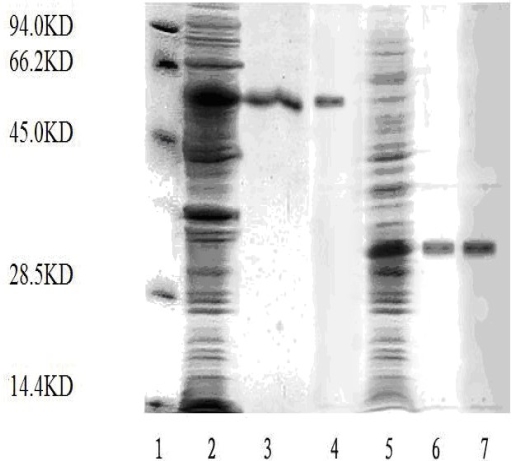
SDS-PAGE and Western-blotting analysis of T591 protein and T1300 protein. 1. Protein molecular mass marker; 2. non-induced T1300; 3. purified T1300; 4. western-blot of purified T1300; 5. non-induced T591; 6. purified T591; 7. western-blot of purified T591.

**Figure 3.**
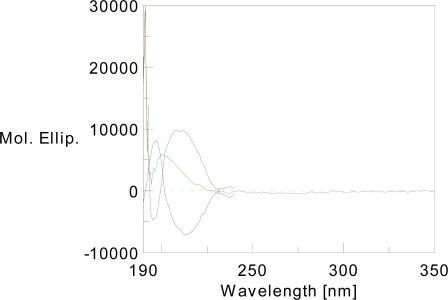
Circular Dichroism analysis of T591 protein.

*E. coli* BL21 transformed pET-28a-T591 or pET-28a-T1300 expressed about 30 KD or 55 KD recombinant protein. The recombinant proteins were detected by SDS-PAGE and Western-blot as described in Materials and Methods (Figure 2). Circular dichroism analysis of T591 revealed the presence of significant secondary structure in the protein (Figure 3). The results indicated that T591 was composed of 32.5% helix, 34.1% β-sheet, 10.2% turn, and 23.4% random structure. Software analysis confirmed the high similarities of the spatial structure between the recombinant protein and the wide-type TACE prodomain.

The recombinant plasmids (pET-28a-T591 and pET-28a-T1300) were constructed successfully. The recombinant proteins (T591 protein and T1300 protein) were expressed and purified. And the purified productions were analyzed by SDS-PAGE and Western blot (The visualization method is coomassie staining).

The circular dichroism can be used to help determine the secondary structure of the prodomain proteins we purified. Molecular modeling software showed that the structure of the recombinant protein is consistent with the predicted structure of the wild-type TACE prodomain.

### 2.2. The Binding of the TACE Prodomain Protein to TACE

The well coated with BSA acted as the control well. As the concentration of T1300 protein increased, the absorbance of the corresponding well increased also. When the concentration of T1300 protein increased to 5 mg/L, the binding reaction was considered positive. The results demonstrated that the prodomain could strongly bind to the TACE catalytic domain (Table 1).

### 2.3. Establishment of the Endotoxemia Model

**Figure 4.**
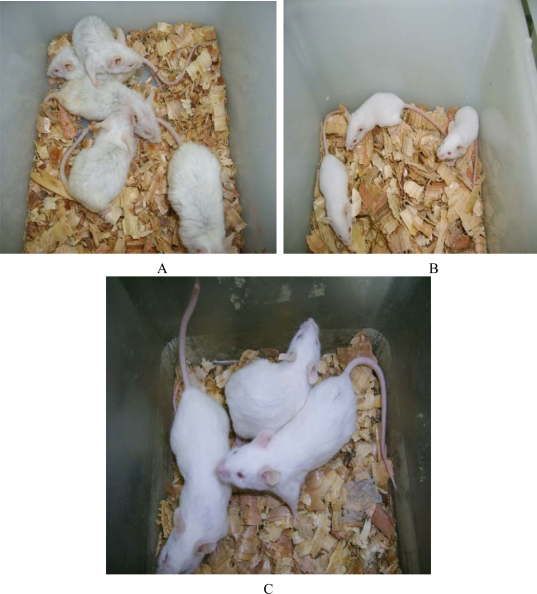
The mice in the endotoxemia model. A. the mice in the LPS group appeared chilled and anxious. B. the mice in the control group. C. the mice in the treatment group (prodomain 50mg/mouse) appeared normol just like those in the control group.

**Figure 5.**
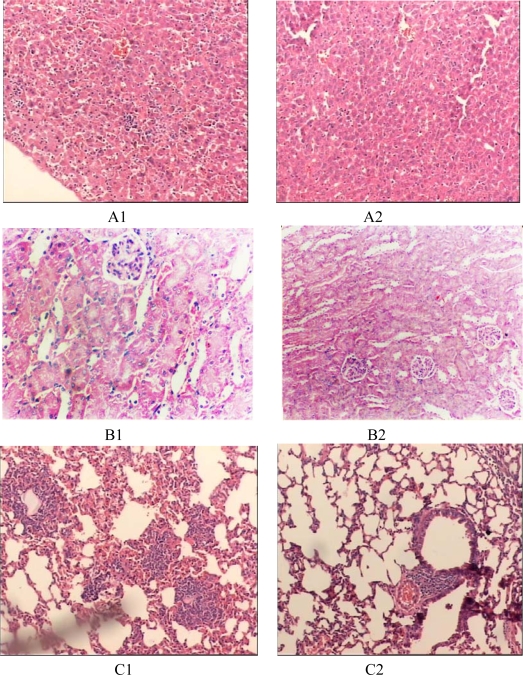
Immunohistochemistry staining of lung, kidnap and liver (HE, original magnification × 200). (A1) liver slice of mice in endotoxic shock model group. (A2) Liver slice of mice in therapic group (20 mg/kg prodomain protein). (B1) kidnap slice of mice in endotoxic shock model group. (B2) kidnap slice of mice in therapic group (20 mg/kg prodomain protein). (C1) lung slice of mice in endotoxic shock model group. (C2) lung slice of mice in therapic group (20 mg/kg prodomain protein).

The mice in the endotoxemia model group had red skin and appeared restless shortly after injection with LPS. Subsequently, 20 minutes after injection, five mice in the LPS group appeared chilled and anxious. Toxic symptoms increased after 40 minutes and included lethargy, diarrhea and cyanosis. In contrast, toxic symptoms in the treated groups were significantly reduced. Mice that were injected with 50 mg of prodomain protein were almost normal, just like those in the control group (Figure 4). HE staining showed the prodomain protein decreased the inflammatory response of liver, kidney and lunges (Figure 5).

HE staining proved the prodomain protein could decrease the inflammatory response of liver, kidney and lung.

### 2.4. The Prodomain Protein Mediated Accumulation of mTNF-α on Murine Peritoneal Macrophages

To further confirm the inhibition of the prodomain protein on TACE, the accumulation of mTNF-α on the surface of murine macrophages was determined by FACS. FACS analysis indicated the expression of mTNF-α in the cure group was higher than that in the endotoxaemia group, and the difference was significant. Also, the mTNF-α expression in the cure group was clearly related to the concentration of the prodomain added (see Table 2). The results demonstrate that the prodomain protein mediated an accumulation of mTNF-α on the cell surface and led to a decrease of sTNF-α release from cells.

With the concentration of the purified T1300 protein increasing, the absorbance of the corresponding well increased. When the concentration of T1300 was greater than 5 mg/L, the absorbance value of the well was more twice than that of the control well coated with BSA, and the reaction of corresponding well was thus considered positive. The results demonstrated that the prodomain could strongly bind to TACE.

The prodomain could inhibit the sTNF-α release from LPS-stimulated macrophage, resulting in accumulation of mTNF-α on the cell surface

## 3. Experimental Section

### 3.1. Construction of Prokaryotic Expression Plasmids of TACE

The cDNA was reverse transcribed with random primers by the Reverse transcription-polymerase chain reaction amplification (RT-PCR) kit (TakaRa, China) using the RNA extracted as template. Reverse transcription conditions were 42 °C for 45 minutes and then 95 °C for five minutes. A portion (2 μL) of the cDNA product was used for subsequent PCR amplification. To amplify the TACE ecotodomain and prodomain gene, nested primers were designed and synthesized according to the published cDNA sequence of TACE. The first version, consisting of the catalytic, disintergrin, and cysteine-rich domains of TACE, referred to as T1300, was amplified with primer 1 (5’-cacggatccttgctgacccagatcccatgaag-3’, containing the *BamHI* site) and primer 2 (5’-ctagaattcctacatcctgtactcgtttctcac-3’, containing the *EcoRI* site). The second version, referred to as T591, encoding the prodomain of TACE, was amplified with primer 3 (5’-gtgggatccccgcgacctccggatgac-3’, containing a *BamHI* site) and primer 4 (5’-ggcgaattctcttttcactcgatgaacaag-3’, containing an *EcoRI* site). Subsequently, the PCR products were purified according to the instruction of the gel extraction kit (TakaRa, China). The purified PCR products (T1300, T591) were cut with *BamHI* and *EcoRI* and subsequently subcloned into the pET-28a(+) prokaryotic expression vector which had been previously digested with the same restriction enzymes to create pET-28a(+)-T1300 and pET-28a(+)-T591. Restriction enzymes and sequencing (Shanghai Bioasis Biotech Co. Ltd, China) were used to confirm the successful construction of the plasmids.

### 3.2. Expression and Purification of TACE Ecotodomain and Prodomain

*E. coli* BL21 (DE3) containing either pET-28a(+)-T1300 or pET-28a(+)-T591 were cultured until *A*_600_ reached 0.3–0.4 then induced with 1 mmol/L IPTC(Sigma, USA) at 37 °C for 6 hours. The proteins were obtained from the inclusion bodies and solubilized in 20 mM Tris-HCL (pH8.0) containing 8 M/L urea and 100 mM/L 2-ME. The solubilized proteins were refolded by a 1:100 dilution in refolding buffer [20 mM/L Tris-HCL (pH7.6) containing 200 mM/L NaCl, 5 mM/L CaCl_2_, 100 mM/L ZnCl_2_, 100 mM/L arginine, and 0.002% NaN_3_]. After incubation for 2 days at 4 °C, the recombinant proteins were purified using a Ni^2+^-chelating resin affinity column. The purified products were analyzed by SDS-PAGE and Western blot.

### 3.3. Circular Dichroism

CD measurements were performed in a JASCO-810 spectropolarimeter. The CD spectra of the T591 protein was recorded at a final concentration of 0.08439 mM/L. The spectra were taken between 190 and 350 nm at room temperature. Scans were done in triplicate. The Insight molecular modeling software was used to analyze the spatial structure of wide-type TACE prodomain.

### 3.4. Binding Analysis of TACE Prodomain Protein to TACE

The activity of T1300 protein can be used to approximate that of wild-type TACE enzyme [20]. So the binding of the prodomain (T591) protein to T1300 protein can reflect the binding of the prodomain to TACE. The binding analysis was performed by ELISA. 96-well polystyrene micro plates were coated with 100 μL 20 mg/L of the prodomain protein or BSA as a control at 4°C overnight. A series of dilutions of T1300 (1 mg/L, 5 mg/L, 10 mg/L, 50 mg/L, 100 mg/L) were then added to the wells coated with prodomain as well as to the control wells and incubated at 37 °C for 30 minutes. Each concentration of T1300 was tested in duplicate well. Each well was washed with rinsing buffer, 100 μL rabbit monoclonal anti-TACE antibody (Sigma, USA) was added, and the mixture incubated for one hour at 37 °C followed by washing three more times. HRP-conjugated anti-rabbit Gig (Sigma, USA) was then added, incubated at 37 °C for 20 minutes and washed with buffer five times. A substrate solution was added to bring about color development. Using a micro plate reader, the absorbance (A_450nm_) of each well was determined. The binding activity of the prodomain protein to TACE was judged by the absorbance of each well. If the ratio between absorbance of the well coated with prodomain and that of the control well was >2, the reaction of the well was considered to be positive.

### 3.5. LPS-Induced Endotoxaemia Model

Female Kunming mice (SPF grade, four weeks old, 15 g to 20 g, from Tongji Medical College, China) were randomly divided into six groups of six animals. Animal care and treatment in this investigation complied with the ARV Statement for the use of Animals in Ophthalmic and Vision Research. Group A, the control group, received normal saline; Group B, the LPS-induced endotoxemia model group, received 0.1 mL solution that contained 10 mg d-galactosamine and 0.4~2 μg LPS, and the solution was injected into the vena caudalis; Group C, D and E were treatment groups, which were received an abdominal cavity injection with the recombinant TACE prodomain protein (group C: 10 mg/mouse; groupD: 20 mg/mouse; group E: 50 mg/mouse; and group F, another control group, received an abdominal cavity injection with prodomain alone (50 mg/mouse).

After 30 minutes, each mouse in the treatment group was injected with the same solution as group B. The response of each mouse was observed, and after 6 h, the mice were sacrificed. Murine peritoneal macrophages were isolated from mice abdominal cavity and the liver, kidney, and lung were removed for traditional histopathology sectioning.

### 3.6. Flow Cytometric Analysis of mTNF-α Level

After washing twice with PBS, the murine peritoneal macrophages were incubated with anti-TNF-α mob (1:100 diluted in PBS, containing 1% BSA) for 1 h at 4 °C. The cells were washed twice with PBS, followed by incubation with FITC-conjugated goat anti-rabbit Gia (1:60 diluted in PBS, containing 1% BSA) for 1 h at 4 °C in the dark. Finally, the cells were washed twice with PBS and resuspended in 0.5 mL PBS until analysis.

### 3.7. Statistical Analysis

Statistical analysis was performed using student’s test. Values were compared between different groups in the experiment. P values < 0.05 were considered statistically significant.

## 4. Conclusions

Many bioactive proteins, including cytokines, growth factors and adhesion molecules, are synthesized as inactive transmembrane precursors that can be released into the surrounding medium upon proteolytic cleavage by specific endoproteinases. Among these protein, the processing and maturation events surrounding tumor necrosis factor-α (TNF-α) have been the most extensively examined to date. The enzyme responsible for TNF-a shedding was isolated in 1997 and is known as TNF-a-converting enzyme (TACE) [13,21]. Sequence analysis confirmed that TACE is a member of the ADAMs (a disintegrin and a metalloproteinase domain family), and is numbered as ADAM17 [22]. TACE cleaves the 26 kD precursor of TNF-α (pro-TNF-α, mTNF-α) to yield the TNF-α soluble form (sTNF-α). Apart from TNF-α, TACE has also been implicated in the ectodomain shedding of many other cell-surface proteins, such as TNFR-1 and TNFR-2.

TNF-α is a cytokine that mediates various inflammation-promoting biological activities. Overproduction of sTNF-α plays a central role in the inflammatory process [10]. To control inflammation and alleviate inflammatory disorders, it is important to reduce the level of sTNF-α or inhibit its function. Because TACE is considered an important sheddase for TNF-α, many attempts have been made to synthesize specific inhibitors to TACE that could ultimately be used to regulate inflammation-related pathologies [23]. None of these compounds, however, have been demonstrated to be TACE-specific. In this paper, we analyzed the structure of TACE to find candidates for specific inhibitors. The ADAM proteases are typically composed of several domains. Besides the enzymically active catalytic domain, an ADAM protease also has an *N*-terminal prodomain as well as several other domains at its C-terminus. The prodomain keeps the metalloprotease site of ADAMs inactive through a cysteine switch [24]. *In vivo*, cleavage of the prodomain is a prerequisite for the generation of an active protease. It is likely that this mechanism of maturation and activation applies to most ADAMs metalloproteases, including ADAM17. The cysteine switch in the TACE prodomain appears to be important for the inhibition of this enzyme, because mutation of the cysteine residue to an alanine or histidine leads to protease activation independent of prodomain cleavage [24]. Thus, the prodomain might act as a potent and specific inhibitor of the catalytic domain of the enzyme.

In this study, we expressed and purified the prodomain protein of TACE. The results of circular dichroism analysis revealed a thermodynamically stable structure for the purified prodomain protein. Furthermore, molecular modeling software showed that the structure of the recombinant protein is consistent with the predicted structure of the wild-type TACE prodomain. Together with the results of binding experiments (Figure 4), we believe that the purified recombinant protein can be used to study the function of the TACE prodomain.

Endotoxemia manifests as a severe clinical syndrome characterized by cytokine release, increased expression of adhesion molecules, release of reactive oxygen species and expression of acute phase proteins. Vascular inflammation occurs within minutes of Gram-negative bacterial infection and coincides with a burst of proinflammatory cytokines derived from activated monocytes and macrophages [25–27]. There is increasing evidence that bacteraemia and endotoxaemia stimulate the immune system into a self-perpetuating, generalized state of hyperactivity. Indeed, injection of LPS into animals reproduces the pathophysiological changes caused by live bacteria, and is considered a standard model for endotoxemia [28,29]. In particular, The LPS induction of sTNF-α activity appears to be a necessary component for LPS-induced bacterial events [30]. Human ADAM17 and its mouse homolog are highly similar, sharing 85% homolog and 91.9% indentity in amino acid sequences. On the basis of this similarity, we choose to reduce the level of sTNF-α or inhibite its function in mice by treatment with the human TACE prodomain prior to the injection of a sublethal dose of LPS. This model was used to test the ability of prodomain to control and alleviate inflammation. We found that endotoxic shock in mice was induced by intravenous injection of LPS resulting in severe pathological changes, such as haemorrhage and necrosis in lung, liver and kidney. Pretreatment of mice with TACE prodomain protein 30 minutes before LPS administration inhibited enzymaic cleavage of mTNF-*α* into sTNF-*α*, causing a marked increase in mTNF-*α* expression of pertioneal macrophages and decrease the inflammatory response of liver, kidney and lunge. These results suggested the prodmain can inhibited the proteolytic activity of ADAM17, since it could inhibit the sTNF-*α* production *in vivo*.

ADAM17 has also been shown to be the major sheddase of several other proteins, including transforming growth factor-*α*, heparin-binding epidermal growth factor-like growth factor, fractalkine, p75 neurotrophin receptor, and MUC1 [31,32]. However, ADAM17 probably does not process all of these substrates simultaneously, and their release from membranes most likely dependent on specific conditions. In this paper, we established an endotoxaemia model, and only investigated the effect of the TACE prodomain on inhibition of the release of sTNF-*α*. Further study is required to determine its effect on other substrates.

In summary, we drew the conclusion that the prodomain is an effective and specific antagonist of TACE, by means of inhibiting the catalytic activity. Inspired by the results, we deduced the TACE prodomain protein has the capability to serve as the effective drugs against inflammation and might be used to cure the TNF-α-related disease.

## Figures and Tables

**Table 1.** The results of ELISA showed the the recombinant pro-domain protein can be binded to TACE.

**Groups of coating antigen**	**Density of coating antigen (mg/L)**	**Density of T1300 (mg/L)**	**OD_450_**
*Pro-domain protein*	20	100	0.69
*Pro-domain protein*	20	50	0.50
*Pro-domain protein*	20	10	0.42
*Pro-domain protein*	20	5	0.28
*Pro-domain protein*	20	1	0.18
*BSA*		100	0.13
*BSA*		50	0.16
*BSA*		10	0.14
*BSA*		5	0.14
*BSA*		1	0.15
*Coating*		10	0.08
*liquor(without antigen)*			

**Table 2.** The recombinant pro-domain protein mediated accumulation of mTNF-α on LPS-stimulated macrophage cells surface.

**Groups**	**mTNF-α expression (%)**
basic control	10.12 ± 8.22
pro-domain alone	9.88 ± 6.35
LPS-stimulation	20.85 ± 10.61
pro-domain protein (10 mg/kg) + LPS	40.34 ± 2.31^a^
pro-domain protein (20 mg/kg) + LPS	94.56 ± 4.11^a^
pro-domain protein (50 mg/kg) + LPS	98.14 ± 2.43^a^

a P < 0.01 compared with LPS-stimulation group.
